# A Deep Look at the Vaginal Environment During Pregnancy and Puerperium

**DOI:** 10.3389/fcimb.2022.838405

**Published:** 2022-05-17

**Authors:** Marco Severgnini, Sara Morselli, Tania Camboni, Camilla Ceccarani, Luca Laghi, Sara Zagonari, Giulia Patuelli, Maria Federica Pedna, Vittorio Sambri, Claudio Foschi, Clarissa Consolandi, Antonella Marangoni

**Affiliations:** ^1^ Institute of Biomedical Technologies – National Research Council, Milan, Italy; ^2^ Microbiology, Department of Experimental, Diagnostic and Specialty Medicine (DIMES), University of Bologna, Bologna, Italy; ^3^ Department of Agricultural and Food Sciences, University of Bologna, Cesena, Italy; ^4^ Family Advisory Health Centres, Ravenna, Italy; ^5^ Unit of Microbiology, Greater Romagna Hub Laboratory, Cesena, Italy

**Keywords:** vaginal microbiome, vaginal metabolome, pregnancy, puerperium, miscarriage, women’s health

## Abstract

A deep comprehension of the vaginal ecosystem may hold promise for unraveling the pathophysiology of pregnancy and may provide novel biomarkers to identify subjects at risk of maternal-fetal complications. In this prospective study, we assessed the characteristics of the vaginal environment in a cohort of pregnant women throughout their different gestational ages and puerperium. Both the vaginal bacterial composition and the vaginal metabolic profiles were analyzed. A total of 63 Caucasian women with a successful pregnancy and 9 subjects who had a first trimester miscarriage were enrolled. For the study, obstetric examinations were scheduled along the three trimester phases (9-13, 20-24, 32-34 gestation weeks) and puerperium (40-55 days after delivery). Two vaginal swabs were collected at each time point, to assess the vaginal microbiome profiling (by Nugent score and 16S rRNA gene sequencing) and the vaginal metabolic composition (^1^H-NMR spectroscopy). During pregnancy, the vaginal microbiome underwent marked changes, with a significant decrease in overall diversity, and increased stability. Over time, we found a significant increase of *Lactobacillus* and a decrease of several genera related to bacterial vaginosis (BV), such as *Prevotella, Atopobium* and *Sneathia*. It is worth noting that the levels of *Bifidobacterium* spp. tended to decrease at the end of pregnancy. At the puerperium, a significantly lower content of *Lactobacillus* and higher levels of *Gardnerella, Prevotella, Atopobium*, and *Streptococcus* were observed. Women receiving an intrapartum antibiotic prophylaxis for Group B *Streptococcus* (GBS) were characterized by a vaginal abundance of *Prevotella* compared to untreated women. Analysis of bacterial relative abundances highlighted an increased abundance of *Fusobacterium* in women suffering a first trimester abortion, at all taxonomic levels. *Lactobacillus* abundance was strongly correlated with higher levels of lactate, sarcosine, and many amino acids (i.e., isoleucine, leucine, phenylalanine, valine, threonine, tryptophan). Conversely, BV-associated genera, such as *Gardnerella*, *Atopobium*, and *Sneathia*, were related to amines (e.g., putrescine, methylamine), formate, acetate, alcohols, and short-chain fatty-acids (i.e., butyrate, propionate).

## Introduction

In healthy reproductive-aged women, the vaginal microbiome is generally dominated by members of the *Lactobacillus* genus (van der Wijgert et al., 2014; Smith and Ravel, 2017). Lactobacilli promote the maintenance of the vaginal health, preventing the colonization and growth of adverse microorganisms through various mechanisms, such as vaginal pH lowering, bioactive compounds production, competition for adhesion, and modulation of immune response (Parolin et al., 2015; Petrova et al., 2015; Foschi et al., 2017). On the other hand, the reduction of lactobacilli combined with the increase of different species of anaerobic bacteria (e.g., *Gardnerella*, *Atopobium*, *Prevotella*, *Mobiluncus*) results in the switch from a normal vaginal ecosystem to a polymicrobial dysbiosis, namely bacterial vaginosis (BV) (Parolin et al., 2018; Ceccarani et al., 2019). This condition is accompanied by marked alterations in the composition of vaginal metabolites, being higher concentrations of various biogenic amines and short chain fatty acids (SCFAs) common fingerprints of BV condition (Yeoman et al., 2013).

On a regular basis, the composition of the vaginal microbiome can vary throughout a woman’s life in response to various factors, such as hormonal status, diet, sexual habits, pharmaceutical treatments, and urogenital infections (Kroon et al., 2018; Noyes et al., 2018; Dall'Asta et al., 2021). Specifically, during pregnancy, the vaginal microbiome undergoes marked changes, with a significant decrease in overall bacterial diversity, an increased stability, and an enrichment of *Lactobacillus* spp. (Aagaard et al., 2012; DiGiulio et al., 2015; Gupta et al., 2020; Marangoni et al., 2021). Contrariwise, in the postpartum period (i.e., puerperium), the vaginal microbiome becomes less *Lactobacillus* spp. dominated, with increased biodiversity (MacIntyre et al., 2015).

It is well known that the composition of the vaginal bacterial communities and related metabolites play a crucial role in maternal-fetal health (Fox and Eichelberger, 2015; Nelson et al., 2016; Laghi et al., 2021). As for healthy vaginal environments, healthy pregnancies are usually characterized by a lactobacilli-dominated ecosystem, whereas reduced lactobacilli abundances, increased bacterial diversity, and higher levels of specific vaginal metabolites (e.g., acetone, formate, isopropanol, methanol) are associated with preterm birth and other complications (Prince et al., 2014; Ansari et al., 2020; Di Simone et al., 2020). For example, reduced prevalence of *Lactobacillus* spp. and higher levels of selected vaginal metabolites (inosine, fumarate, xanthine, benzoate, ascorbate) (Al-Memar et al., 2020; Xu et al., 2020; Marangoni et al., 2021) seem to be predictors of a higher risk of spontaneous miscarriage.

In this context, only few studies have investigated the association between the structure of the vaginal ecosystem and the first trimester miscarriage (Zhang et al., 2019; Al-Memar et al., 2020; Fan et al., 2020; Xu et al., 2020), while many aspects about the dynamic interactions between the inhabitants of the vaginal ecosystem, their metabolites, and the host remain to be fully elucidated despite the recent advances in the study of the human microbiome during pregnancy and puerperium (Vinturache et al., 2016; Gupta et al., 2020).

Therefore, the aim of this study was to deepen the characteristics of the vaginal environment in a cohort of Caucasian women with a normal pregnancy throughout their different gestational ages (i.e., first, second, third trimester) and puerperium. A group of women suffering a spontaneous first trimester miscarriage was also included for a wider characterization. For each subject and each time point, both the vaginal bacterial composition (16S rRNA sequencing) and the vaginal metabolic profiles (Proton nuclear magnetic resonance spectroscopy-^1^H-NMR) were analyzed.

## Materials and Methods

### Study Cohort and Samples Collection

Subjects were enrolled among all the Caucasian pregnant women presenting to the Family Advisory Health Centers of Ravenna (Italy) for prenatal care starting from April 2019.

Exclusion criteria were the following: (i) age < 18 years; (ii) HIV positivity; (iii) body mass index (BMI) > 33; (iv) medically assisted procreation; (v) use of any antibiotics in the month preceding the sampling; (vi) use of vaginal douches or topical agents in the two weeks before sampling; (vii) presence of uncontrolled chronic diseases (e.g., diabetes, autoimmune disorders, malignancies); (viii) drug addiction or heavy smokers (> 15 cigarettes/day). Moreover, women with urogenital infections due to sexually transmitted pathogens (i.e., *Chlamydia trachomatis*, *Neisseria gonorrhoeae, Trichomonas vaginalis, Mycoplasma genitalium*), aerobic vaginitis (AV) or symptomatic vulvo-vaginal candidiasis (VVC) were further excluded after the laboratory testing.

At gestational age of 9-13 weeks (first trimester), 20-24 weeks (second trimester), 32-34 weeks (third trimester), and puerperium (40-55 days after delivery) women underwent an obstetric examination. For all patients, demographic data and information about urogenital symptoms were recorded.

Women colonized with Group B *Streptococcus* (GBS) at the third trimester of pregnancy received intrapartum antibiotic prophylaxis (IAP) (i.e., penicillin G or ampicillin), following international guidelines (Kolkman et al., 2020).

Two vaginal swabs were collected at each time point. The first one (E-swab, Copan, Brescia, Italy) was used for microbiological diagnostic tests and Nugent score assessment. The second was collected with a sterile cotton bud, re-suspended in 1 mL of sterile saline, and stored at -80°C until use. Frozen vaginal swabs were thawed, vortexed for 1 min and removed from the liquid. After centrifugation (10000 × *g* for 15 min), the cell-free supernatants were used for metabolomic analysis, whereas bacterial pellets were employed for vaginal microbiome profiling.

A written informed consent was obtained from all subjects and the study protocol was approved by the Ethics Committee of Romagna (CEROM) (n° 2032 of 21^st^ February 2018). This study was carried out in accordance with the Declaration of Helsinki, following the recommendations of the Ethics Committee.

### Microbiological Investigations

A commercial nucleic acid amplification technique (NAAT) was used for *C. trachomatis*, *N. gonorrhoeae*, *T. vaginalis* and *M. genitalium* detection (Seeplex STI Master Panel 1; Seegene, Seoul, KR). VVC was excluded by the microscopic presence of fungal buds and a significant growth of *Candida* colonies by culture (Yano et al., 2019). AV were diagnosed by means of a microscopic examination (i.e., diminished/absent lactobacilli, presence of leukocytes, parabasal cells, small coliform bacilli, cocci, or chains), combined with the growth of aerobic microorganisms, mainly of intestinal origin, by culture (Donders et al., 2011).

A Gram stain scoring system (Nugent score) was used for a preliminary assessment of the vaginal flora composition (Nugent et al., 1991). Based on this score, women were grouped as follows: ‘H’ group (normal lactobacilli-dominated microbiota, score 0-3), ‘I’ group (intermediate microbiota/flora, score 4-6), ‘BV’ group (bacterial vaginosis, score 7-10) (Zozaya-Hinchliffe et al., 2010).

### Vaginal Microbiome Profiling

Nucleic acids were extracted from vaginal swabs by means of the Versant molecular system (Siemens Healthcare Diagnostics, Tarrytown, NY, USA) equipped with a sample preparation module designed for automated sample preparation (Marangoni et al., 2015).

The V3-V4 hypervariable regions of the bacterial 16S rRNA gene were amplified, according to the 16S metagenomic sequencing library preparation protocol (Illumina, San Diego, CA, USA). Final indexed libraries were prepared by equimolar (4 nmol/L) pooling, denaturation, and dilution to 6 pmol/L before loading onto the MiSeq flow cell (Illumina). A 2 × 300 bp paired-end run was used.

Raw reads were analyzed according to a previously described procedure (Severgnini et al., 2021). Briefly, a single fragment from two overlapping pairs was generated using PandaSeq software (v2.5, Masella et al., 2012). Downstream analyses, such as filtering, zero-radius Operational Taxonomic Units (zOTUs) creation, taxonomy assignments, and diversity analyses were performed using the QIIME suite (release 1.9.0, Caporaso et al., 2010), unoise3 algorithm (Edgar, 2016), RDP classifier (Wang et al., 2007), and SILVA 16S rRNA database (release 132, https://www.arb-silva.de/fileadmin/silva_databases/qiime/Silva_132_release.zip).

Characterization of *Lactobacillus* spp. was performed by BLAST-aligning all reads belonging to that genus to a custom reference database made up collecting all available reference sequences in NIH-NCBI database ftp://ftp.ncbi.nlm.nih.gov/genomes/GENOME_REPORTS/prokaryotes.txt of 17 *Lactobacillus* species commonly found in the vaginal environment, with finishing status of “complete genome”, “chromosome” or “scaffold”. Potential matches were filtered in order to retrieve an unequivocal classification for each read, according to the procedures already described (Ceccarani et al., 2019, **Supplementary Material**). Since 2020, *Lactobacillus* taxonomy underwent major update, with the re-classification of the genus in 25 different genera (23 of which are novel) (Zheng J. et al., 2020). Old and new species names used in the present article are available as **Supplementary Table S1**.

Alpha-diversity evaluation was estimated according to several microbial diversity metrics (i.e., chao1, Shannon index, observed species, Good’s coverage, and Faith’s phylogenetic distance). Beta-diversity analysis was conducted using both weighted and unweighted Unifrac metrics (Lozupone et al., 2011), and through the Principal Coordinates Analysis (PCoA).

### Metabolomic Analysis

Metabolomic analysis was performed by means of a ^1^H-NMR spectroscopy starting from 700 µL of the cell-free supernatants of the vaginal swabs, added to 100 μL of a D_2_O solution of 3-(trimethylsilyl)-propionic-2,2,3,3-d4 acid sodium salt (TSP) 10 mM set to pH 7.0 (Foschi et al., 2018)

^1^H-NMR spectra were recorded at 298 K with an AVANCE III spectrometer (Bruker, Milan, Italy) operating at a frequency of 600.13 MHz, equipped with Topspin software (Ver. 3.5) according to previously described procedures (Foschi et al., 2018). The signals originating from large molecules were suppressed by a CPMG filter of 400 spin-echo periods, generated by 180° pulses of 24 μs separated by 400 μs (Ventrella et al., 2016).

To each spectrum, line broadening (0.3 Hz) and phase adjustment was applied by Topspin software, while any further spectra processing, molecules quantification and data mining step was performed in R computational language (version 4.1.2., R Core Team, 2021) by means of in-house developed scripts.

The spectra were aligned towards the right peak of alanine doublet, set to 1.473 ppm. The spectra were then baseline-adjusted by means of peak detection according to the “rolling ball” principle (Kneen and Annegarn, 1996) implemented in the “baseline” R package (Liland et al., 2016). A linear correction was then applied to each spectrum, to make the points pertaining to the baseline randomly spread around zero.

The signals were assigned by comparing their multiplicity and chemical shift with Chenomx software data bank (ver 8.3, Chenomx Inc., Edmonton, Alberta, Canada). Quantification of the molecules was performed in the first sample acquired by employing the added TSP as an internal standard. To compensate for differences in sample amount, any other sample was then normalized to such sample by means of probabilistic quotient normalization (Dieterle et al., 2006). Integration of the signals was performed for each molecule by means of rectangular integration.

### Data Analysis and Statistics

Statistical evaluation of α-diversity indices was performed by non-parametric Monte Carlo-based tests through the QIIME pipeline. Beta-diversity differences were assessed by a permutation test with pseudo F-ratios using the “adonis” function from R package “vegan” (version 2.0-10, Oksanen et al., 2013). Pairwise relative abundance analysis was performed using a non-parametric Mann–Whitney U test. For comparing relative abundances across multiple categories, we applied a Kruskal-Wallis test, followed by Dunn’s *post-hoc* test for pairwise comparisons.

Metabolite concentrations were correlated to bacterial composition by calculating Spearman’s correlation coefficient between metabolites and bacterial genera present ≥1% in at least 1 sample (n=51). In this analysis, we considered all data points at T1, T2, T3, and T4. We performed a Spearman’s rank-based correlation between genus relative abundances and metabolite quantities, selecting only those with p-value< 0.05 (i.e.: correlation significantly different from 0). To better visualize patterns of positively correlated bacteria and metabolites, a heatmap was drawn, clustering correlation coefficients for metabolites and bacteria (using Pearson’s correlation as clustering metric and average linkage).

For each statistical analysis, unless otherwise stated, p-values < 0.05 were considered as significant. Statistical analyses were performed using MATLAB software (Natick, MA, USA).

### Data Availability

Raw sequencing data of 16S rRNA gene are available at NCBI Short-reads Archive (SRA) with BioProject accession number PRJNA766806 (https://www.ncbi.nlm.nih.gov/sra/PRJNA766806). Raw metabolomic data are available as a **Supplementary material (Data Sheet S1)**.

## Results

### Study Population

A total of 63 Caucasian pregnant women with a mean age of 30.8 **±** 5.1 years (min: 21, max: 44) and a mean BMI of 23.5 ± 3.5 (min: 16.0, max: 32.5) were enrolled and sampled during all gestational ages; for 30 of them, clinical and microbiological data were also available for the puerperium.

In addition, 9 women (mean age: 33.8 ± 6.4 years; mean BMI: 24.5 ± 4.7) who had a spontaneous miscarriage at the first trimester of pregnancy (gestational age: 11-13 weeks) during the study were included. From the first to the third trimester of pregnancy, we noticed a significant decrease of BV cases, together with an increase of samples characterized by a normal microbiota (*p*=0.001; **Table 1**). During the puerperium, only one third of the women (33.3%) showed a lactobacilli-dominated flora, being most of them characterized by an alteration of the vaginal bacterial composition (26.6% intermediate flora, 40.0% BV-condition).

**Table 1:** Characteristics of the vaginal environment (Nugent score), stratified by the different gestation periods and conditions.

	First trimester miscarriage (n=9)	Normal pregnancies (n=63)			Puerperium (n=30**)
		1^st^ trimester	2^nd^ trimester	3^rd^ trimester	
**Nugent score** **0-3 (H)** **4-6 (I)** **7-10 (BV)**	22.2% (2/9) 55.5% (5/9) 22.2% (2/9)	49.2% (31/63) 33.3% (21/63) 17.5% (11/63)	74.6% (47/63) 15.9% (10/63) 9.5% (6/63)	82.5% (52/63) 11.1% (7/63) 6.4% (4/63)	33.3% (10/30) 26.6% (8/30) 40.0% (12/30)
		*p=*0.001*			

H, lactobacilli-dominated microbiome; I, intermediate flora; BV, bacterial vaginosis.

^*^statistical significance is referred to the differences in microbial composition (Nugent score) during the three trimesters of pregnancy, excluding women suffering a first trimester miscarriage.

^**^clinical and microbiological data during the puerperium were available only for 30 women.

Ten out of the 30 (33.3%) women with puerperium data available received an intrapartum antibiotic prophylaxis to prevent GBS neonatal infection.

Most cases of spontaneous abortion were associated with an altered vaginal microbiome (55.5% intermediate status (I); 22.2% BV condition).

### Vaginal Microbiome Structure Characterization

Overall, microbiota composition assessed through 16S rRNA sequencing was in accordance with what expected for the vaginal environment, with the *Lactobacillus* genus having an average relative abundance of 77.9%, followed by *Gardnerella* (8.9% on average), *Bifidobacterium* (3.5%), *Atopobium* (2.1%), *Prevotella* (1.8%), and *Megasphaera* (1.3%). Other genera, such as *Sneathia, Ureaplasma, Aerococcus*, and *Dialister* had <1% abundance. The first 12 genera accounted for 97.7% of the overall relative abundance, confirming the relatively low biodiversity of the vaginal samples (**Supplementary Figure S1**).

**Figure 1:**
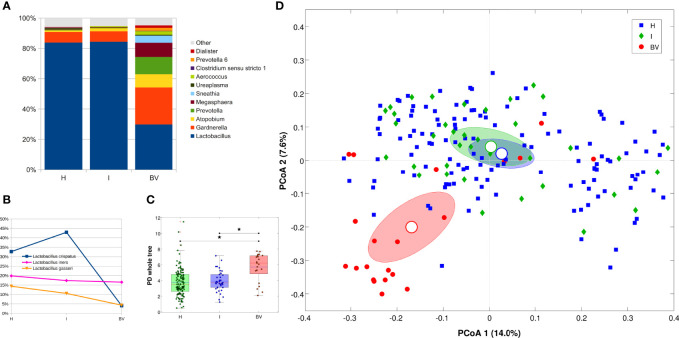
Microbiota characterization according to the vaginal status (H, I or BV). **(A)** Barplot of average relative abundances at genus level. Genera with rel. ab. ≤1% were grouped in “Others” category; **(B)** Line plot of average *Lactobacillus* species abundance per vaginal status; only the 3 most abundant species are represented; **(C)** Boxplot of Faith’s phylogenetic diversity of the samples (estimated at endpoint) for each vaginal status. Stars above the plots represent statistical significance (p<0.05); **(D)** Principal Coordinate Analysis (PCoA) based on unweighted Unifrac distance among samples. Each point represents a sample; ellipses are 95% SEM-based confidence intervals; point and ellipses are grouped according to vaginal status; the first and the second coordinate are represented.

Microbiota structure was evaluated according to the vaginal status derived from the Nugent score, for a total of 189 samples (63 women at 3 time points each), comparing healthy (H) with intermediate (I) and bacterial vaginosis (BV) status. As expected, BV condition was characterized by a profound alteration of the microbiota, with a dramatic reduction of *Lactobacillus* spp. (83.8% vs 29.8% for H and BV, respectively) and an increase of opportunistic bacteria, (i.e., *Gardnerella*, *Atopobium*, *Prevotella*, *Megasphaera*, *Sneathia*, *Aerococcus*). The I condition seemed to be composed by nearly the same bacterial members of the H samples, with little exceptions in low-abundant members of the community (i.e., *Dietzia, Actinomyces, Enterococcus, Cutibacterium,* and ‘*Eubacterium eligens group*’), all contributing with <0.2% of average abundance (**Figure 1A**). Among the lactobacilli species, a significant reduction of *L. crispatus* was highlighted in BV samples as compared to both H and I (avg. abundance: 4.1% BV vs 32.7% H, 42.9% I); on the other hand, *L. iners* (avg. abundance: 19.9% H, 17.4% I, 16.5% BV), as well as other *Lactobacillus* species, was not affected (**Figure 1B**). Differences in microbial composition were reflected in α-diversity analysis, which highlighted a significant increase in biodiversity in BV samples as compared to H and I ones (*p*=0.003 for all metrics) (**Figure 1C**). Moreover, we recorded a significant separation of microbial profiles (β-diversity) among BV, I, and H (*p*<0.039 for all pairwise group comparisons, unweighted Unifrac); at the same time, BV samples differed from the other two groups in major contributors of the microbiota (*p*=0.001 against both H and I conditions, weighted Unifrac) (**Figure 1D**). Similarly, distances between H and BV samples were higher than H vs I ones and I vs BV distances were higher than H vs I (both for weighted and unweighted Unifrac); at the same time, weighted Unifrac distances among BV samples resulted higher than that among H or I samples, confirming that BV status was characterized by a deeper alteration of the microbial composition with respect to other conditions.

### Taxonomic Composition of the Vaginal Bacterial Communities During Pregnancy

We evaluated the vaginal microbiota dynamics along the three trimesters of pregnancy for a total of 63 women (189 total samples). The proportion of samples with the same vaginal status at each trimester was found to be statistically different for the H subjects (*p*<0.001, two-sided proportion test without continuity correction; increasing from 49.2% to 74.6% and 82.5% respectively at T1, T2, and T3) and the I subjects (*p*=0.005; decreasing from 33.3% at T1 to 15.9% at T2 and 11.1% at T3). On the other hand, no differences were highlighted for BV status (proportion of 17.5%, 9.5%, and 6.3% respectively at T1, T2, and T3).

There were no significant or noticeable differences in biodiversity over time, other than T1 vs T2 in chao1 (*p*=0.039) and T1 vs T3 for the Faith’s phylogenetic diversity metric (*p*=0.015). With regards to microbial composition, T3 points were statistically separated from T1 and T2 sets (*p ≤* 0.015, unweighted Unifrac), which were indistinguishable from each other; no differences on the weighted Unifrac distance matrix were highlighted (**Supplementary Figures S2A, B**).

Analyzing bacterial genera co-abundance patterns, we were able to identify four co-abundance groups (CAGs) (**Supplementary Figure S3**): (i) *Ureaplasma* alone; (ii) *Lactobacillus* CAG (also including *Clostridium*); (iii) ‘opportunistic’ bacteria CAG (including *Bifidobacterium*, *Prevotella* and *Dialister*); (iv) BV-associated bacteria CAG (i.e.: *Gardnerella*, *Atopobium*, *Megasphaera*, *Sneathia* and *Aerococcus*). At all three time points, *Lactobacillus* CAG was inversely correlated to other CAGs, whereas opportunistic and BV-related CAGs were directly associated to one another, although with a different strength of correlation.

**Figure 2:**
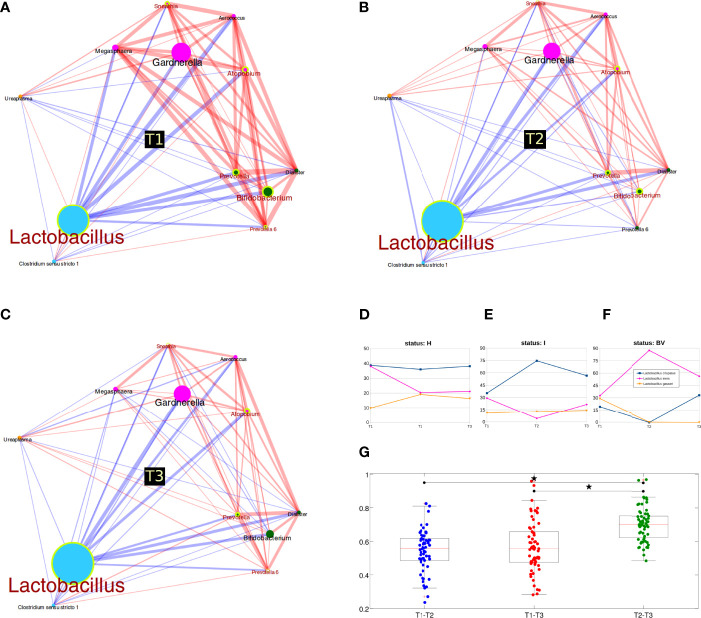
Microbiota evolution during three trimesters of pregnancy. **(A–C)** Co-abundance networks of bacterial genera over time. Circle size is proportional to genus relative abundance for each time and colors are according to co-abundance groups (see also Suppl_Figure3_heatmap_CAGnetwork); edge size is proportional to the strength of correlation; red lines mean positive correlation, while blue lines indicate negative correlation. Genera resulting statistically different over time points are highlighted with a yellow circle and a red label; **(D–F)** *Lactobacillus* species abundance over time, stratified for vaginal status. Only the three most abundant species are represented; **(G)** Boxplot of unweighted Unifrac distances between samples over time. Distances were calculated for each pair of samples belonging to the same women, sampled at T1, T2 or T3; stars above the plots represent statistical significance (p<0.05).

Many genera were statistically different over time, suggesting a deep reshaping of the microbiota between the first two trimesters: all groups were differential in both T1 vs T2 and T1 vs T3 comparisons, but not for T2 vs T3. In particular, we revealed increased *Lactobacillus* abundances and reduced levels of opportunistic (such as *Bifidobacterium* and *Prevotella*) and BV-related bacteria (*Atopobium* and *Sneathia*) (**Figures 2A–C**). Stratification by the vaginal status allowed a deeper evaluation of changes over time: in BV samples, we highlighted a shift between T1 and T2 among the phyla *Actinobacteria* (increased) and *Fusobacteria* (decreased), while at genus level *‘Prevotella (group 6)’* was found decreased; for the I condition, no major members of the microbiota were statistically different among pregnancy trimesters (we observed only a total of 6 differential genera over time, all with average relative abundance <0.3%); finally, in the H samples, we observed a reduction of the phylum *Actinobacteria* and of its related genus *Bifidobacterium* (avg. rel. ab. of 7.5%, 2.1%, and 3.2% respectively at T1, T2, T3); the *Bifidobacterium* reduction was also confirmed when limiting the analysis to the 22 women with a “healthy” microbiota (H group) at each pregnancy time-point (avg. rel. ab. 8.4%, 1.6%, and 1.0% respectively at T1, T2, T3). The *Streptococcus* genus was also decreased in the H group (entirely taken), but with a consistently lower abundance (avg. rel. ab. 0.5%, 0.1%, 0.2% respectively for T1, T2, and T3) (**Supplementary Figures S2C**).

As for evaluations within the *Lactobacillus* species, stratifying for the vaginal status, we observed several variations. In the H group, we highlighted the slight (non-significant) reduction of *L. iners* and the increase of *L. gasseri* in T1 vs T2, whereas abundances were nearly identical for T2 and T3; on the other hand, *L. crispatus* abundances were fundamentally unaltered. Considering the I group, a significant reduction of proportion of *L. iners* and a significant increase of *L. crispatus* was observed between T1 and T2. BV samples had the opposite trend, with a reduction of *L. crispatus* and an increase of *L. iners* between T1 and T2 (**Figures 2D–F**). No differences in *Lactobacillus* species were highlighted considering all samples together, regardless of their vaginal status.

Lastly, we analyzed the Unifrac distances among samples. The first interesting evidence was that the microbial profiles of all samples collected from one woman were more similar to each other than to those collected from the other women (*p*<0.001, intra- vs inter-distance for both weighted and unweighted Unifrac). When evaluating distances over time, we recorded that T2 vs T3 distance (unweighted Unifrac) was significantly higher than both T1 vs T2 and T1 vs T3 (comparisons not significantly different), indicating that between the second and third trimester of pregnancy the microbiota develops in a more independent way (**Figure 2G**). Considering the H samples alone, the evolution between T2 and T3 was confirmed; furthermore, our result suggests that T3 represents an evolution of the microbiota from T2 (as T1 had lower distance values to T2 than to T3), although average distances were very similar (T1-T2: 0.68, T1-T3: 0.71, T2-T3: 0.71).

### Vaginal Microbiome at the Puerperium

In addition, we sought to evaluate the vaginal microbiome characteristics during the puerperium period in a group of 30 women, sampled a fourth time during the study (total subset time-points: T1, T2, T3 during pregnancy; T4 at puerperium, 40-55 days after delivery).

We did not observe a different biodiversity over time (*p*>0.05 for all α-diversity metrics tested); on the other hand, there seemed to be some separation in microbial composition within the β-diversity analysis, as T4 points were statistically different from T1, T2, and T3 (both unweighted and weighted Unifrac) (**Supplementary Figure S4A**). Over time, the analysis of microbial relative abundances at genus level suggested a composition variation at T4, with a lower content of *Lactobacillus* and a consistent presence of *Gardnerella*, *Prevotella*, *Atopobium*, and *Streptococcus*; those changes were significant (*p*<0.05) when compared to T1 (all except *Atopobium* and *Gardnerella*), to T2 (all except *Gardnerella*), and to T3 (all genera) (**Supplementary Figure S4B**). At species level, this was reflected in a significant decrease of all *Lactobacillus* species, *L. crispatus* and *L. jensenii* in particular (*p*<0.05), while *L. gasseri* was also found decreased but not significantly; contrariwise, *L. iners* was observed to be unchanged during the puerperium.

**Figure 3:**
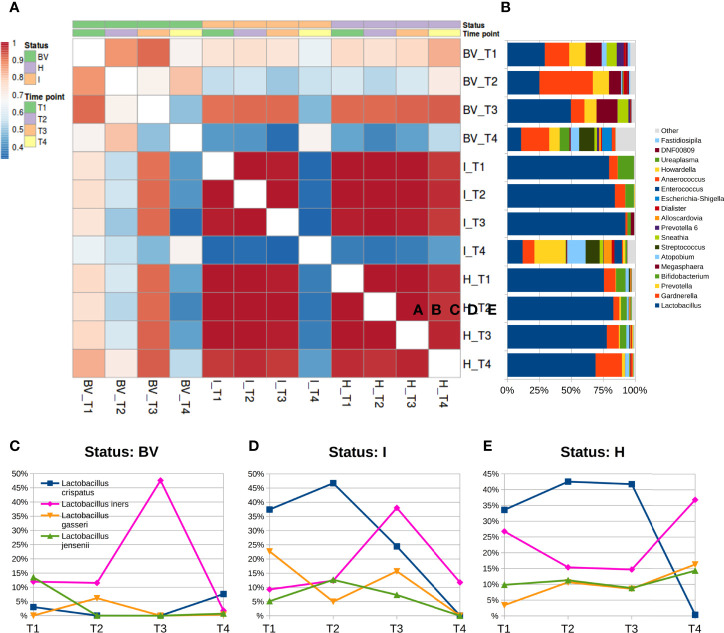
Microbiota evolution during puerperium (T4). **(A)** Heatmap of Pearson’s correlation coefficients calculated between average relative abundances at genus level over time and stratified for vaginal status; **(B)** barplots of average relative abundances at genus level over time and stratified for vaginal status; genera with rel. ab. ≤1% were grouped in “Others” category; **(C–E)** Line plots of average abundances of *Lactobacillus* species over time. Only the four most abundant species are represented.

The stratification by vaginal status highlighted how these differences were mainly due to a change in bacterial members for the BV and I groups, whereas microbial profiles of H women resulted more stable, as evidenced by analyzing the correlation coefficients among average microbial profiles at genus level over time. Within the H group, microbial composition did not vary consistently, with an average Pearson correlation between T4 and all of the other three time-points of r=0.977, similar to the average r=0.998 between the paired comparisons of T1, T2, and T3; on the other hand, correlation coefficients for BV women were lower and slightly different over time (r=0.659 between T4 and the other three time points; r=0.826 among T1, T2, and T3); finally, the most substantial differences were observed for the I group, as the correlation coefficient dropped from r=0.994 to r=0.340 when comparing T4 to the other three time points (**Figures 3A, B**). BV samples at T4 were characterized by a significant reduction of the genera *Lactobacillus*, *Megasphaera*, and *Prevotella*, and by an increase of *Streptococcus* and *Finegoldia* with respect to T1; we observed a significant reduction of *Lactobacillus* and an increase of *Prevotella*, *Streptococcus*, and *Dialister* for the I condition in the comparison of T4 to all other gestational time-points; despite a slightly increase in the *Gardnerella* abundance, microbial profiles of the H group resulted very similar over all four time-points.

The *Lactobacillus* species analysis stratified by the vaginal status suggested that BV samples at puerperium had a switch compared to both the first and second trimester: *L. crispatus* showed a higher abundance (T4 7.6% vs T1 3.0% and T2 <0.1%; *p*<0.05 for T2 vs T4) while *L. iners* a lowered one (T4 1.8% vs T1 12.0% and T2 11.5%; *p*<0.05 for T1 vs T4). We could not evaluate the BV composition during the third trimesters (T3) since we had only 1 sample for this time-status combination. In the I samples, T4 microbiota displayed a dramatic decrease of *L. crispatus* (0.1% vs 36.2% of the gestational time-points average; *p*<0.05 forT2 vs T4). A similar decrease of *L. crispatus* was observed for H women as well (0.4% *vs* 39.3% on average of the gestational time points), together with a somewhat higher abundance of *L. iners* (36.8% *vs* 19.0% of the gestational time-points average). Due to the extreme variability among individuals, between T3 and T4 the sole *L. crispatus* reduction was statistically significant (**Supplementary Figures S3C–E**).

Among the women evaluated at T4, 10 out 30 (33.3%) received an intrapartum antibiotic prophylaxis for GBS. Microbial profiles of these women did not result significantly different from the untreated group (n=20) neither by alpha- (*p*>0.05 for all metrics tested) nor beta-diversity (*p*=0.937 and *p*=0.112 for unweighted and weighted Unifrac distances, respectively). Only one taxon, the *Prevotella* genus, was significantly altered, showing an increase in antibiotic-treated women, when compared to the untreated ones (rel. ab. of 20.0% with antibiotics vs 6.0% without antibiotics) (**Supplementary Figure S5**). At the same time, this difference was reflected in higher-level taxonomies as well (*Bacteroides*, *Bacteroidia*, *Bacteroidales*: 22.9% vs 8.0%; *Prevotellaceae*: 22.2% vs 7.3%, with vs without antibiotics). No differences were highlighted by the species-level characterization of the *Lactobacillus* genus.

### Association Between Microbiome Composition and First Trimester Miscarriage

We compared the microbiome profiles at T1 of the 63 women with successful pregnancies to the ones of 9 women who suffered a first trimester miscarriage. No significant differences were found on both α- (*p*>0.05 for chao1, Shannon, Good’s coverage, Observed species, Faith’s phylogenetic diversity metrics) and β-diversity (*p*=0.412 and *p*=0.110 for unweighted and weighted Unifrac distances, respectively) analyses. Nevertheless, we observed an overgrowth of *Fusobacterium* (rel. ab. 1.1%, *p*=0.02) in the miscarriage group compared to successful pregnancies (0.1%). No significant differences were highlighted for the *Lactobacillus* species.

### Vaginal Metabolites Composition and Metabolite-Microbiome Correlation

In the supernatants of the vaginal swabs, a total of 63 metabolites were detected and quantified by ^1^H-NMR spectroscopy. Molecules mainly belonged to the groups of SCFAs, organic acids, amino acids, and biogenic amines (Data sheet S1).

We performed a correlation analysis aimed at relating microbial composition to metabolite concentrations, using Spearman’s rank correlation to determine monotonically increasing or decreasing relationships. All samples collected over the four time-points were considered (n=219); miscarriage samples were analyzed separately.

**Figure 4:**
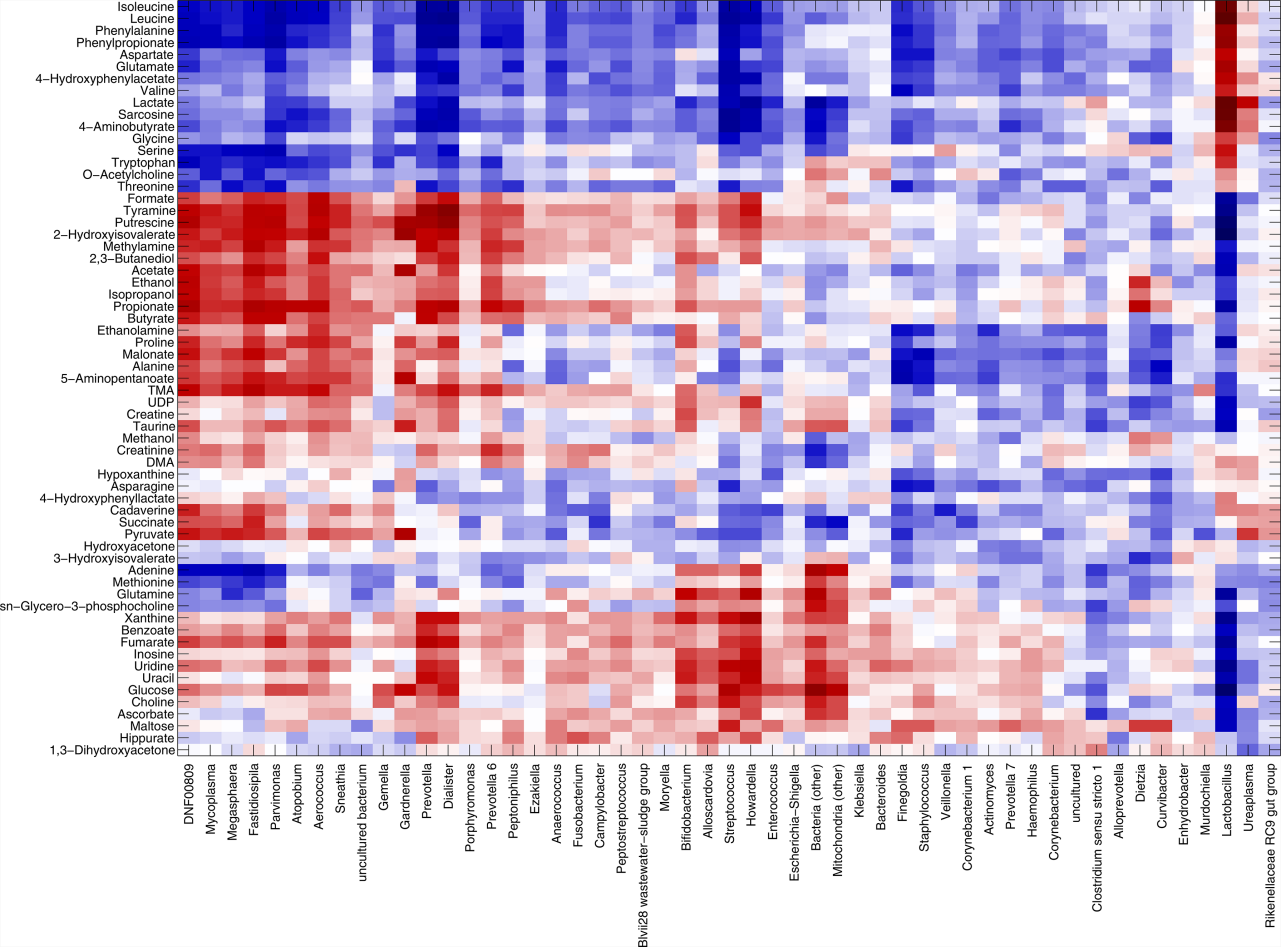
Correlation between metabolome and microbiota. Heatmap showing the Spearman’s correlation coefficient between metabolites concentration and the relative abundances of the main bacterial genera over all samples collected, excluding miscarriages (n=219). Only groups present at >1% of relative abundance in at least one sample were considered. Metabolite and microbial data were clustered using Pearson’s correlation metric and average linkage.

We could define three main clusters of correlations: (i) *Lactobacillus* stood by itself, separated from all other bacteria, strongly positively correlated to lactate and sarcosine (r=0.62 and r=0.61, respectively). Moreover, positive correlations were evidenced for many amino acids (i.e., isoleucine, leucine, phenylalanine, aspartate, glutamate, valine, glycin, serine, threonine, tryptophan, with correlation values ranging from 0.26 to 0.65); (ii) BV-associated genera, such as *Gardnerella*, *Prevotella*, *Atopobium*, *Dialister*, *Aerococcus*, and *Sneathia*, were positively correlated to putrescine, methylamine, tyramine, formate, trimethylamine (TMA), alcohols (i.e., ethanol, isopropanol), and SCFAs (i.e., acetate, butyrate, propionate); (iii) other lower-abundance bacteria, such as *Bifidobacterium*, *Streptococcus*, and *Alloscardovia* correlated with nucleotides (i.e., adenine, glutamine, inosine, uracil), glucose, choline, benzoate, and fumarate (**Figure 4**).

Microbiome-metabolites correlation patterns were further refined by looking at the possible relationships with spontaneous miscarriages (n=9). Overall, only few correlations were significant (p-value of the linear model <0.05). Correlation patterns for *Lactobacillus* and BV-associated genera were in accordance with those described above for the samples of women with a successful pregnancy. Interestingly, *Fusobacterium* was found positively correlated to the nucleotides and their components (i.e., uracil, adenine, UDP, tyramine, r range 0.36-0.56), as well as to methionine (r=0.65), formate (r=0.54), choline, xanthine, and maltose (r=0.43-0.49), putrescine (r=0.38), and methylamine (r=0.31) (**Supplementary Figure S6**).

## Discussion

A deep comprehension of the vaginal ecosystem may hold promise for unraveling the pathophysiology of pregnancy and may provide novel markers to identify women at risk of complications, such as miscarriage and preterm births. Moreover, considering that microbial communities can be transferred from the mother’s vaginal niche to the newborn gut, the study of the vaginal microbiome during pregnancy and puerperium can open new perspectives for infant’s microbiome development and future health (Dominguez-Bello et al., 2010).

That is why in this study we characterized the vaginal environment in the situations of both a normal pregnancy, at the three gestational trimesters and the puerperium period, and a spontaneous first trimester miscarriage. In particular, we assessed the vaginal bacterial composition and the vaginal metabolic profiles.

At first, we confirmed that, irrespective of the period and type of pregnancy, BV cases were characterized by a dramatic reduction of *Lactobacillus* and an increase of anaerobic bacteria, such as *Gardnerella, Atopobium, Prevotella, Megasphaera, Sneathia*, *Aerococcus* (Deka et al., 2021).

In line with previous findings, the relative and absolute proportion of *L. crispatus*, a hallmark of vaginal eubiosis, inclined to decrease in the transition from H to BV conditions (Ceccarani et al., 2019). As for *L. iners*, the abundance of this species did not differ between H and BV groups in our cohort, even though it has been considered a transitional species typically associated with dysbiotic conditions (Yoshimura et al., 2020); on the other hand, *L. iners* has also been reported to be the dominating taxon in a large subset of women worldwide, being its presence associated with young age and unprotected sexual practices (France et al., 2020; Novak et al., 2021).

When considering the changes of the vaginal microbiome during the three trimesters of pregnancy, we observed that several bacterial genera were statistically different between the first and second trimester, suggesting a deep reshaping of the microbiome profiling towards a ‘healthier condition’: moving from the first to the third trimester, in line with the higher proportion of H cases, we found an increase of *Lactobacillus* genus and a decrease of BV-related genera (e.g., *Prevotella, Atopobium*, *Sneathia*), with no differences in *Lactobacillus* species.

Taken together, these data confirmed that the vaginal microbiome becomes more stable throughout the entire pregnancy, being less diverse and mainly dominated by lactobacilli (Li et al., 2020; Rasmussen et al., 2020; Marangoni et al., 2021; Pace et al., 2021).

It is worth noting that bifidobacteria, typical beneficial commensals inhabiting the human intestine, have tended to decrease their vaginal ecosystem abundances at the end of pregnancy. It has been shown that *Bifidobacterium* is the dominant genus of some vaginal microbiomes and that overall bifidobacteria have the potential to be as protective as lactobacilli, according to the current understanding of a healthy vaginal microbiome (Freitas and Hill, 2017). Nevertheless, Lee and colleagues recently observed that the relative abundance of *Bifidobacterium* spp. significantly increased during pregnancy in women with an intermediate and BV status compared to normal vaginal microbiota, and that some dysbiotic conditions were dominated by *Bifidobacterium breve* (Lee et al., 2020). In line with these observations, our data highlighted a co-abundant vaginal pattern, characterized by several BV-associated genera, such as *Prevotella* and *Dialister*, and *Bifidobacterium* spp. Since the role of this vaginal microbial group is yet to be understood, further studies are needed to investigate the clinical significance of the bifidobacteria reduction at the end of pregnancy, as well as to assess the potential impact on newborn’s health (Bozzi et al., 2018).

Other interesting data emerged when looking at the vaginal environment after delivery. In agreement with previous reports (Nunn et al., 2021), at the puerperium we found a significantly lower content of *Lactobacillus*, and higher levels of *Gardnerella, Prevotella, Atopobium*, and *Streptococcus* compared to the third trimester of pregnancy. These variations are consequences of after-delivery vaginal alterations that profoundly altered the host environment and, thus, led to changes in different bacterial species survival and proliferation capabilities (Nunn et al., 2021).

Moreover, we observed a significant increase in *Prevotella* abundance in women who received an intrapartum antibiotic prophylaxis (IAP) for GBS compared to untreated ones. This aspect deserves attention considering that members of *Prevotella* genera have been associated with negative ‘outcomes’ of the cervicovaginal environment, being responsible for strong inflammatory conditions, cytotoxicity, and alterations of the reproductive tract (Campisciano et al., 2020; Salliss et al., 2021). It is well known that IAP can negatively affect the gut microbiome of infants vaginally delivered, specifically in relation to microbial composition and occurrence of antibiotic resistance genes (Garcia, 2021). However, the effect of antibiotic prophylaxis on the vaginal microbiome after delivery is still little explored. Even if further studies are needed to clarify the reasons behind the increase in *Prevotella* levels in women receiving IAP, we can speculate that beta-lactam antibiotics could have selected this bacterial genus, as it is potentially able to produce β-lactamase enzymes (Toprak et al., 2020).

Moving to the analysis of bacterial relative abundances in women suffering a first trimester miscarriage, we highlighted a significant vaginal overgrowth of *Fusobacterium* in abortions compared to successful pregnancies, at all taxonomic levels. This microbial genus has been strongly associated with genital inflammation and dysbiosis, being *Fusobacterium* able to cooperate with other taxa to disrupt the normal vaginal bacterial composition, leading to microbial imbalance (Lennard et al., 2017; Agarwal et al., 2020). It has been shown that *Fusobacterium* has a mutualistic relationship with the BV-correlated bacteria: as they are major sialidase-producers, they enable *Fusobacterium* to consume sialic acids from the host-produced mucus. At the same time, *F. nucleatum* exposure to vaginal communities may encourage features of dysbiosis (e.g., increased sialidase activity and *G. vaginalis* abundance) in susceptible vaginal communities (Agarwal et al., 2020). In addition, *F. nucleatum* has been previously associated with preterm labor, since it was found in greater abundance in preterm placental membranes than at term (Doyle et al., 2014). To the best of our knowledge, this is the first time that *Fusobacterium* got linked to the risk of first trimester miscarriage, considering that previous investigations highlighted the potential role of other microorganisms, such as *Finegoldia, Coprococcus, Roseburia, Atopobium*, and *Prevotella* (Al-Memar et al., 2020; Xu et al., 2020; Jiao et al., 2021; Liu et al., 2021).

The vaginal bacterial community profiles found during pregnancy were accompanied by peculiar fingerprints in the composition of the vaginal metabolites. In agreement with recent observations, *Lactobacillus* abundance was strongly correlated with higher levels of lactate, sarcosine, and many amino acids, whereas BV-associated genera, such as *Gardnerella, Atopobium*, *Sneathia*, were correlated to amines (putrescine, methylamine, TMA), formate, alcohols (ethanol, isopropanol), and short-chain fatty-acids (SCFAs, as butyrate, acetate, propionate) (Ceccarani et al., 2019; Laghi et al., 2021). On the one hand, the lactate production by *Lactobacillus* species reduces the vaginal pH, contributing to the homeostasis against potentially endogenous or exogenous pathogens. These microorganisms are also known producers of branched-chain amino acids, thus the higher concentration of some of them, such as valine, leucine, and isoleucine, is another fingerprint of the prevalence of lactobacilli in ‘healthy’ women (Vitali et al., 2015). Conversely, during dysbiotic conditions, the proliferation of diverse bacterial genera, some of which typical of the gut microbiota, and the imbalance between lactobacilli and BV-related bacteria lead to higher levels of amines, organic acids, and SCFAs (Vitali et al., 2015). In this context, higher levels of *Fusobacterium*, associated with the higher risk of spontaneous abortion, were positively correlated to several vaginal molecules, including methionine, formate, putrescine, and methylamine. Considering the low number of data points (n=9), the exact role of the vaginal metabolome in first trimester miscarriages, as well as the causative relationship between microbiota and immune responses, remain to be further elucidated, to enable the best possible diagnosis and therapeutics of early pregnancy loss.

In conclusion, we deepened the existing literature knowledge about the composition of the vaginal ecosystem during pregnancy and puerperium, highlighting peculiar microbial/metabolic fingerprints.

Our data could help implement ‘prognostic’ criteria (e.g., evaluation of the risk of spontaneous miscarriage based on the microbiome/metabolome profiles), as well as strategies for the prevention of early pregnancy loss, based on the ‘manipulation’ of the vaginal bacterial inhabitants (e.g., use of probiotics and prebiotics). Moreover, the microbial changes induced by GBS prophylaxis (i.e., increase in *Prevotella* levels) deserve attention, leading to the idea of new approaches able to reduce the impact of antibiotics in maternal/neonatal health.

As a strength of our work, we excluded from the enrollment all the women harboring conditions able to perturb per se the vaginal microbiome composition (e.g., VVC, AV, presence of STIs) and we combined multiple ‘omic’ sciences (i.e., genomic and metabolomic) to decipher the vaginal environment in pregnancy and puerperium. On the other hand, we are fully aware of some limitations of the study, as the potential loss of low concentration molecules, due to the reduced sensitivity of ^1^H-NMR compared to other metabolomic techniques (e.g., high resolution chromatographic separation techniques coupled to accurate tandem mass spectrometry).

To further understand the interactions between vaginal microbes and the host, future studies perspectives will include (i) the increase in number of women suffering a spontaneous first trimester miscarriage, to strengthen the conclusions regarding this group (ii) the evaluation of several inflammatory markers, (iii) the assessment of vaginal proteomic profile, and (iv) the evaluation of bacterial subspecies/clades.

## Data Availability Statement

The original contributions presented in the study are publicly available. This data can be found here: National Center for Biotechnology Information (NCBI) BioProject database under accession number PRJNA766806.

## Ethics Statement

The studies involving human participants were reviewed and approved by the Ethics Committee of Romagna (CEROM) (n° 2032 of 21st February 2018). The patients/participants provided their written informed consent to participate in this study.

## Author Contributions

AM and CF conceived and designed the study. SZ and GP recruited the patients. LL, MP, SM, MS, CCo, CCe, and TC performed the experiments. CF, LL, MS, CCo, CCe, and TC analyzed the data. AM and VS contributed reagents/materials and analysis tools. CF, AM, and MS wrote the paper. All authors contributed to the article and approved the submitted version.

## Funding

This study was supported by ‘Fondazione del Monte di Bologna e Ravenna’ (Prot. N°329bis/2017). The funder had no role in study design, data collection and analysis, decision to publish, or preparation of the manuscript.

## Conflict of Interest

The authors declare that the research was conducted in the absence of any commercial or financial relationships that could be construed as a potential conflict of interest.

## Publisher’s Note

All claims expressed in this article are solely those of the authors and do not necessarily represent those of their affiliated organizations, or those of the publisher, the editors and the reviewers. Any product that may be evaluated in this article, or claim that may be made by its manufacturer, is not guaranteed or endorsed by the publisher.
