# Supplementary material for: A Deep Look at the Vaginal Environment During Pregnancy and Puerperium
Source: Front Cell Infect Microbiol. 2022 May 17;12:838405. doi: 10.3389/fcimb.2022.838405 (PMC9152327; doi:10.3389/fcimb.2022.838405)

SUPPLEMENTAL INFORMATION

A deep look at the vaginal environment during pregnancy and puerperium

Marco Severgnini[1†], Sara Morselli[2†], Tania Camboni[1], Camilla Ceccarani[1], Luca Laghi[3], Sara Zagonari[4], Giulia Patuelli[4], Maria Federica Pedna[5], Vittorio Sambri[2,5], Claudio Foschi[2*], Clarissa Consolandi[1§], Antonella Marangoni[2§]

[1]Institute of Biomedical Technologies – National Research Council, Segrate, Milan, Italy

[2]Microbiology, DIMES, University of Bologna, via Massarenti 9, Bologna, Italy

[3]Centre of Foodomics, Department of Agro-Food Science and Technology, University of Bologna, P.za Goidanich, 60, Cesena, Italy

[4]Family Advisory Health Centres, via Berlinguer 11, Ravenna, Italy

[5]Unit of Microbiology, Greater Romagna Hub Laboratory, Piazza della Liberazione 60, Pievesestina di Cesena, Italy

† Marco Severgnini and Sara Morselli equally contributed to this research article

§ Clarissa Consolandi and Antonella Maragnoni equally supervised this research article

* To whom correspondence should be addressed: claudio.foschi2@unibo.it

SUPPLEMENTARY TABLES.. 3

Table S1. Table of the species used for Lactobacillus species-level classification..3

SUPPLEMENTARY FIGURES.. 4

Figure S1. Overall microbiota composition...4
Figure S2. Microbiota characterization during pregnancy..5
Figure S3. Co-abundance groups (CAGs) definition..6
Figure S4. Microbiota characterization in puerperium..7
Figure S5. Effect of antibiotics treatment on microbiota...8
Figure S6. Correlation between metabolome and microbiota for miscarriages...9

SUPPLEMENTARY TABLES

Table S1. Table of the species used for Lactobacillus species-level classification.
Due to the re-classification of the Lactobacillus genus in 25 different genera, the 17 interesting species for the vaginal environment were divided in “Taxonomy changed” or “Taxonomy unchanged”. In case of re-classification, new and old species names are provided.

Taxonomy changed[a]		
**Old name**	**New name[b]**	**Taxonomy unchanged**
Lactobacillus fermentum	*Limosilactobacillus fermentum*	*Lactobacillus iners*
Lactobacillus plantarum	*Lactiplantibacillus plantarum*	*Lactobacillus crispatus*
Lactobacillus brevis	*Levilactobacillus brevis*	*Lactobacillus gasseri*
Lactobacillus casei	*Lacticaseibacillus casei*	*Lactobacillus jensenii*
Lactobacillus vaginalis	*Limosilactobacillus vaginalis*	*Lactobacillus acidophilus*
Lactobacillus salivarius	*Ligilactobacillus salivarius*	*Lactobacillus delbrueckii*
Lactobacillus reuteri	*Limosilactobacillus reuteri*	*Lactobacillus helveticus*
Lactobacillus rhamnosus	*Lacticaseibacillus rhamnosus*	*Lactobacillus johnsonii*
Lactobacillus paracasei	*Lacticaseibacillus paracasei*	*Lactobacillus iners*

[a] Classification according to Zheng J., Wittouck S., Salvetti E. et al., (2020). A taxonomic note on the genus Lactobacillus: Description of 23 novel genera, emended description of the genus Lactobacillus Beijerink 1901, and union of Lactobacillaceae and Leuconostocaceae. https://doi.org/10.1099/ijsem.0.004107

[b] Correspondences between new and old names was determined thanks to “Lactotax” webservice: http://lactotax.embl.de/wuyts/lactotax/

SUPPLEMENTARY FIGURES

Figure S1. Overall microbiota composition.

Barplots of relative abundances at genus level for the samples collected from 63 women during three trimesters of pregnancy (n=189). Genera with an average relative abundance <0.25% were grouped in "Other" category

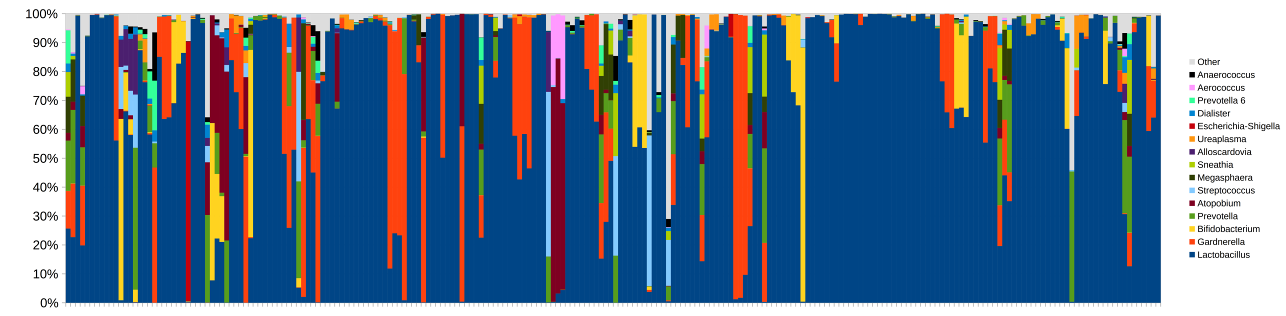

Figure S2. Microbiota characterization during pregnancy.

(A) Boxplots of alpha-diversity values according to four different metrics over time; (B) Principal Coordinate Analysis based on unweighted Unifrac distance among samples. Each point represents a sample; ellipses are 95% SEM-based confidence intervals; point and ellipses are grouped according to time point; the second and third coordinate are represented; (C) Barplots of average relative abundances at genus level over time, stratified according to the vaginal status. Only the 14 most abundant genera are represented.

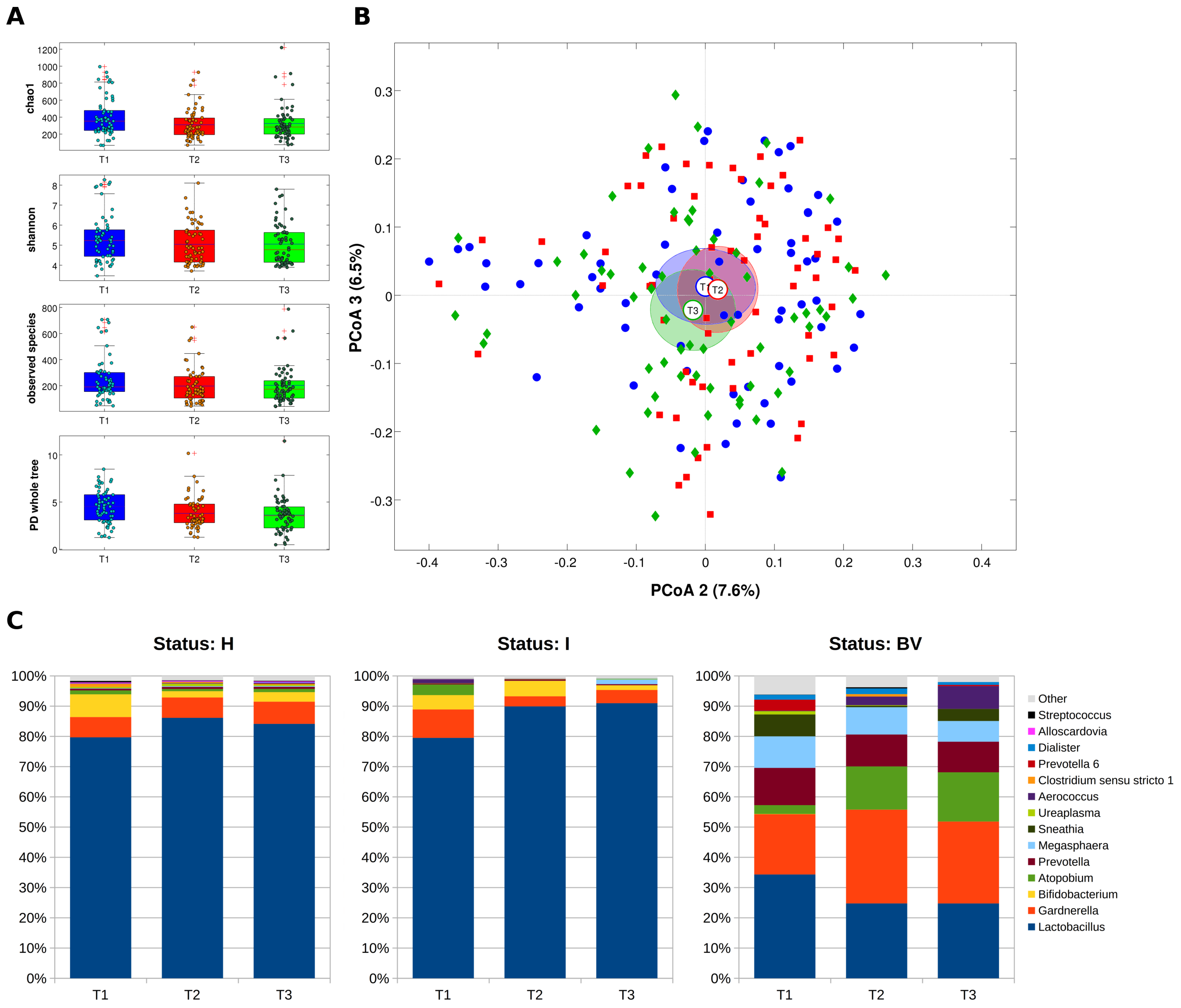

Figure S3. Co-abundance groups (CAGs) definition.

Correlation heatmap among bacterial genera used for determining CAGs. Spearman's rank-based correlation was employed and genera were hierarchically clustered according to Spearman’s correlation metric and Ward linkage. The cut-off at four CAGs was determined empirically. Statistical analysis via multi-group ANOVA confirmed that clusters were significantly different (p<0.05, see also Figure 2).

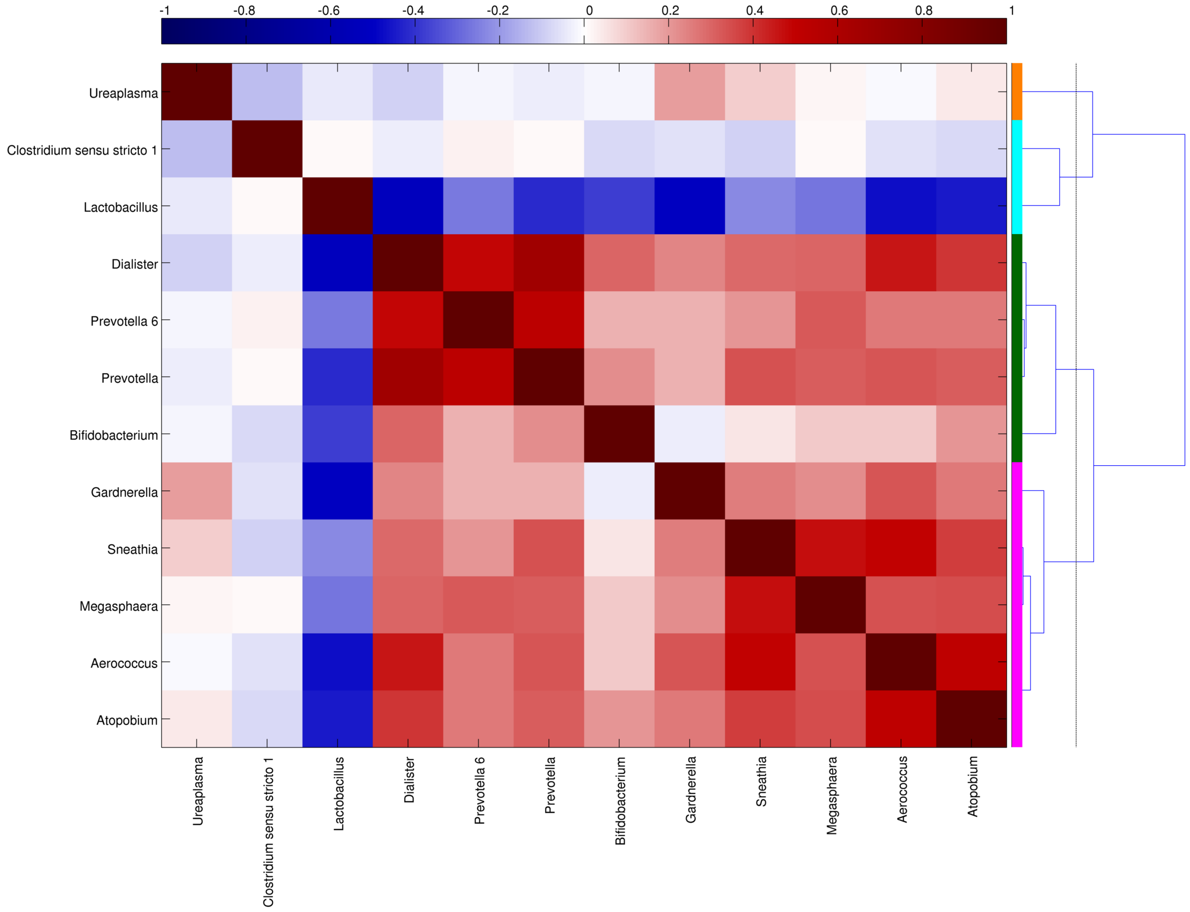

Figure S4. Microbiota characterization in puerperium.

(A) Principal Coordinate Analysis based on unweighted Unifrac distance among samples. Each point represents a sample; ellipses are 95% SEM-based confidence intervals; point and ellipses are grouped according to time point; the first and second coordinate are represented; (B) Boxplots of relative abundances at genus level over time. The first six genera differential in at least one comparison are represented.

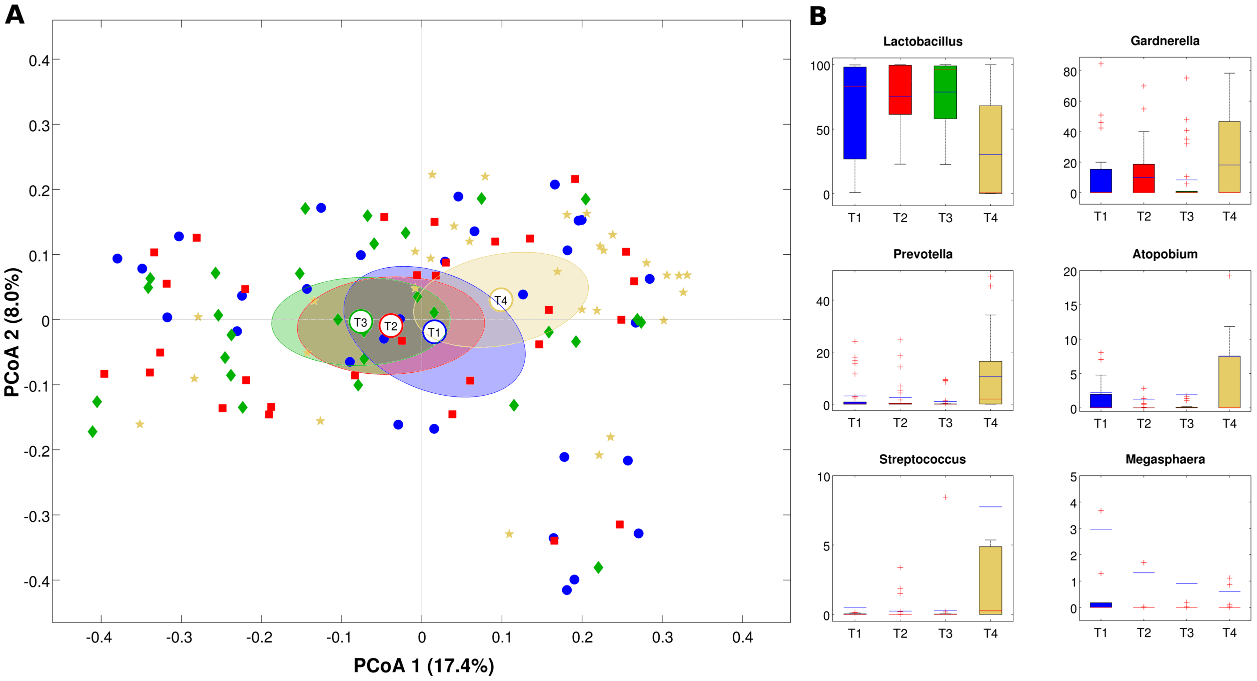

Figure S5. Effect of antibiotics treatment on microbiota.

Pie charts of average microbial composition at genus level for samples taken from women treated with antibiotic prophylaxis to prevent GBS infection (n=10) and untreated ones (n=20). For graphical reasons, only the thirteen most abundant genera are represented.

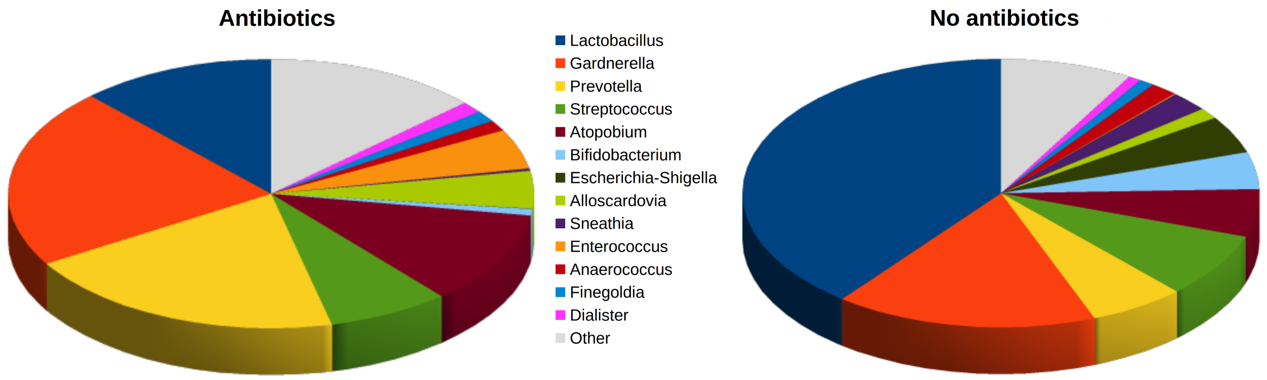

Figure S6. Correlation between metabolome and microbiota for miscarriages.

Heatmap showing the Spearman's correlation coefficient between metabolites concentration and the relative abundances of the main bacterial genera over samples from women who underwent first-trimester miscarriage (n=9). Only groups present at >1% of relative abundance in at least one sample were considered. Metabolite and microbial data were clustered using Pearson's correlation metric and average linkage.

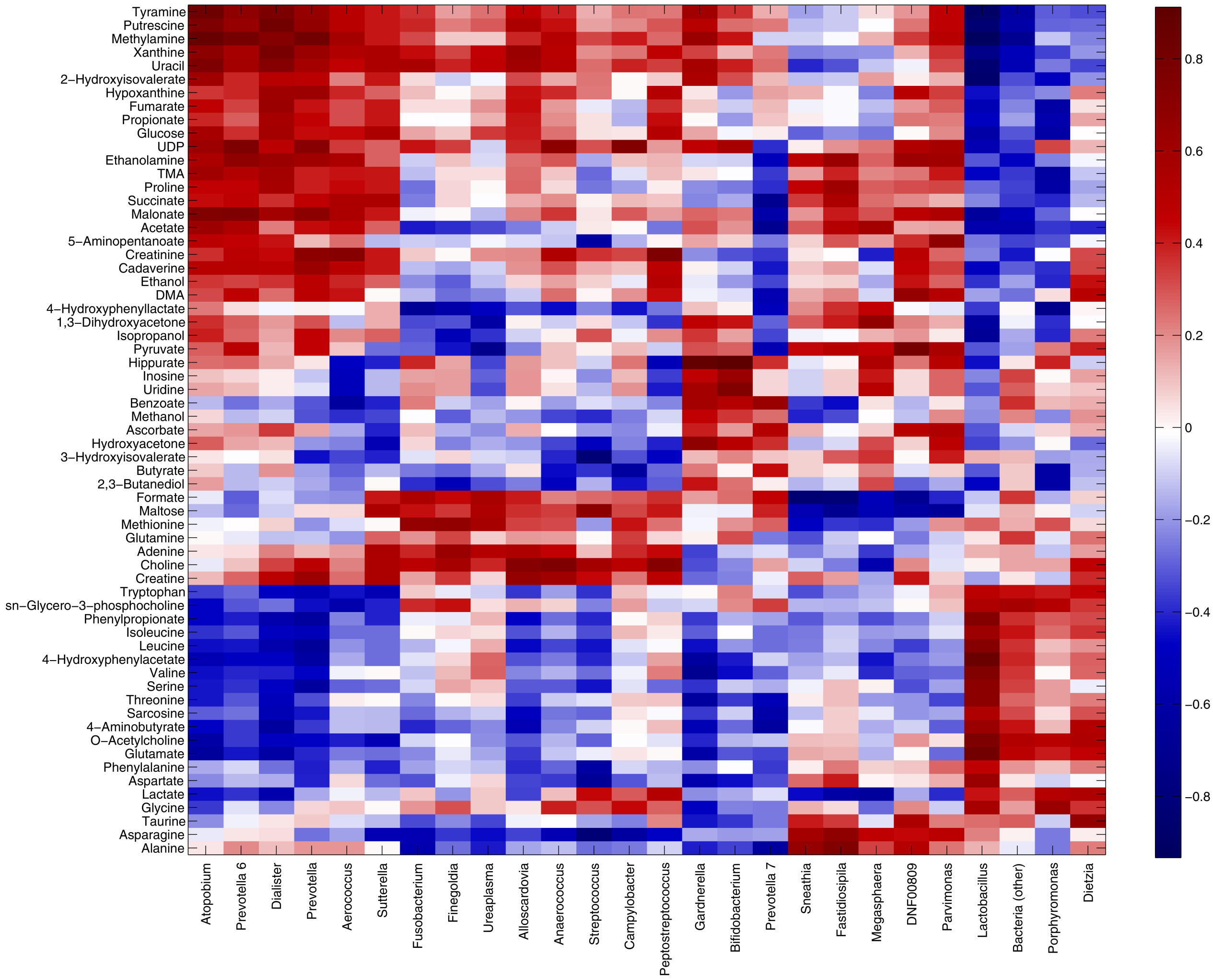

Supplement: Supplementary file 2 [file Presentation_1.pdf]
